# Cricopharyngeus muscle dysfunction: a poorly defined disorder from diagnosis to treatment

**DOI:** 10.1007/s00405-024-08644-7

**Published:** 2024-05-07

**Authors:** Brittany N. Krekeler, Rebecca J. Howell

**Affiliations:** 1 Department of Otolaryngology-Head & Neck Surgery, University of Cincinnati College of Medicine, Cincinnati, USA; 2 Department of Neurology and Rehabilitation Medicine, University of Cincinnati College of Medicine, Cincinnati, USA; 3 Department of Communication Sciences and Disorders, University of Cincinnati College of Allied Health Sciences, Cincinnati, USA

**Keywords:** Laryngology, Cricopharyngeus, Swallowing, Treatment

## Abstract

**Purpose:**

Cricopharyngeus muscle dysfunction (CPMD) is a common clinical occurrence with very few clear diagnostic criteria and multiple pathways for treatment. Incidence of CPMD is not known, but some data suggest around 25% of people with dysphagia experience some degree of CPMD, which negatively impacts swallowing safety and efficiency. Workup and treatment of CPMD can require multidisciplinary collaboration across laryngologists, speech-language pathologists with training in dysphagia management, and gastroenterologists. The purpose of this paper is to review what is known about CPMD and identify areas of future research in CPMD diagnosis and treatment.

**Methods:**

An overview of CPMD, relative treatments and disorders, and a discussion of future areas of research needed to improve clinical care of CPMD.

**Results:**

Details regarding historical background, pathophysiology and treatment practiced for CPMD are included.

**Conclusion:**

In summary, CPMD is a poorly defined disease due to a lack of understanding of its pathophysiology and the lack of consensus diagnostic criteria. Well-designed, prospective clinical trials are necessary to develop a better understanding of clinical incidence of CPMD, impact of the disorder on oropharyngeal swallowing, and how to approach treatment of the disorder surgically or in conjunction with therapy directed by a specialized speech-language pathologist.

## Introduction

Normal or functional swallowing, also referred to as deglutition, involves the transit of a food or liquid bolus from the oral cavity to the stomach via the pharynx and esophagus. Any disruptions of bolus transit through these anatomical regions results in abnormal swallowing or dysphagia. Dysphagia often refers to both biomechanics underlying abnormal bolus transit and patient symptomology related to sensation of abnormal transit. Using anatomic boundaries, we attempt to specifically describe dysphagia based on the region of biomechanical or physiological disturbances in swallowing, including oral, pharyngeal, esophageal, or a combination (e.g. oropharyngeal or pharyngoesophageal dysphagia). Oropharyngeal dysphagia typically involves dysfunction of a combination of oral (i.e. tongue) and pharyngeal (i.e. hyolaryngeal complex) musculature. Pharyngoesophageal dysphagia is characterized by dysfunction of the pharyngoesophageal segment (PES), defined superiorly by the middle and inferior constrictors, the cricopharyngeus muscle (CP), and inferiorly by the circular and longitudinal esophageal muscles.

The cricopharyngeus (CP) muscle is a sphincteric group of fibers contributing to the high-pressure zone known as the upper esophageal sphincter (UES) contained within the PES. Dysfunction in the CP musculature, called cricopharyngeus muscle dysfunction (CPMD), is a highly relevant clinical issue, as CMPD can degrade both swallow safety and efficiency, and cause patients to be highly symptomatic [1]. Prevalence of CP dysfunction is unknown, as differential diagnosis of CPMD requires a combination of instrumentation via videofluoroscopy and/or manometric measures with obstructive symptoms. No recent literature exists estimating clinical prevalence; however, one radiologic study suggests 25% of patients in a sample of over 800 patients undergoing swallowing videofluoroscopy had cricopharyngeus dysfunction [2].

CPMD typically presents clinically as globus sensation. It is difficult to diagnose without appropriate instrumentation and is more likely underdiagnosed as patients present in a multitude of settings including ambulatory, inpatient, nursing and rehabilitative facilities. The workup of dysphagia often includes a multidisciplinary team that may include gastroenterologists, thoracic/foregut surgeons, pulmonologists, intensivists, primary care, with special attention to the roles of speech-language pathology and otolaryngology. While it is commonly cited that CPMD can negatively impact swallowing physiology and patient-reported outcomes, [3–5] overall impact of CPMD on safety and efficiency is poorly defined in the literature. Treatment strategies for CPMD are diverse and include both therapeutic management with speech-language pathologists and surgical management with otolaryngologists. The purpose of this review is to highlight what is currently known about cricopharyngeus muscle function and dysfunction, both in biological and physiological presentation, discuss current strategies for treating CPMD, and highlight the many remaining gaps in the evidence regarding best practice for CPMD management.

## Historical background

Valsalva was the first to describe the inferior portion of the inferior constrictor as the cricopharyngeus muscle in 1717 [6]. Next, physician Abraham Ludlow in 1769 describes the first case of an “obstructed deglutition from a preternatural dilatation and bag formed in the pharynx” [7]. Pathologist Friedrich Albert von Zenker with physician Hugo Wilhelm von Ziemssen collaborated on dilatations of the esophagus in 1878 [8]. Gustav Killian, a laryngologist, was the first to describe the cricopharyngeus muscle as the constrictor of the upper esophagus in 1908 [9]. The anatomic boundaries of the pharyngoesophageal segment were illustrated by Edward Bald Jamieson in his textbook the Illustrations of Regional Anatomy [10]. Two English radiologists Rolf Olsson and Olle Ekberg in 1995 first described the radiologic findings today known as a cricopharyngeus bar (CP bar) [11]. In 1976, Palmer first postulated that a CP bar could be caused by achalasia, spasm, or hypertrophy [12, 13]. Today, unfortunately many of these terms are used interchangeably with CPMD, rather than describing more specific causes of CPMD.

## Cricopharyngeus muscle function

The upper esophageal sphincter, containing the cricopharyngeus musculature, is positioned superior to the esophagus. The purpose of this high-pressure zone is to prevent backflow of swallowed material into the pharynx. In healthy people, during a swallow the hyolaryngeal complex rises and moves anteriorly, and in a combination with pressures driven by base of tongue and pharyngeal wall movement, assists in relaxation of the CP segment to allow bolus passage [14]. The esophageal wall is composed of four layers: mucosa, submucosa, muscularis propria, and adventitia. The upper esophageal sphincter region, where the cricopharyngeus is contained, is a musculocartilaginous structure composed of the cricoid plate, hyoid bone, cricopharyngeus, thyropharyngeal (i.e. inferior pharyngeal constrictor), and cranial cervical esophagus. The cricopharyngeus muscle inserts into the latter part of the cricoid cartilage in a C-shaped configuration where the cricoid plate is anteriorly oriented [15]. Muscle fiber orientation in this region is also distinct; the cricopharyngeus is striated muscle with mostly slow-twitch oxidative fibers with 40% connective tissue, facilitating sustained contraction between swallows [15, 16]. This striated muscle which forms the muscularis propria of the upper esophagus and the UES is formed from the mesenchymal layer of the 4th, 5th, and 6th branchial arches. The UES innervation comes from the vagus nerve, a 5th branchial arch derivative and the recurrent laryngeal nerve, the 6th branchial arch nerve, [15] with motor neuron control housed in the brainstem (nucleus ambiguous of medulla oblongata) [17]. The UES does not exhibit the constant neurogenic or myogenic tone; instead low-level activity of the UES occurs at baseline. In fact, inter-swallow UES pressures are sufficiently low (6–18 mmHg) suggesting that the elastic properties of the CP rather than active contraction contribute to the tone of the UES at rest and during sleep [18]. The UES is controlled by a variety of reflexes from a wide array of stimuli from the pharynx, esophagus, airway, and central nervous system to allow contraction or relaxation of the tonic UES (see Table 1 cited from Kahrilas [19]). UES contraction, and to a lesser degree relaxation, are also subject to volitional control as demonstrated with high-resolution pharyngeal manometry (HRPM) [20].

**Table 1:** Physiological variables and reflexes known to modulate intraluminal UES pressure (cited from Kahrilas [60])

Elicit UES contraction	Elicit UES relaxation
Primary or secondary peristalsis	Swallowing
Slow esophageal/UES distension	Belching
Pharyngeal stimulation with air or water	Rapid esophageal air distension
Respiration (contracts, either in or out of phase)	Sleep
Pulmonary distention (Hering-Breuer reflex)	Sedation/anesthesia
Coughing	Retching and vomiting
Sneezing	Volitional control
Emotional stress	
Volitional control	

The relaxation variables highlighted in bold are associated with sufficient UES opening to result in the antegrade or retrograde flow of air, liquid, or solid food. Opening requires either sufficiently high intraluminal esophageal pressure to overcome residual UES tone or an active assist from the hyolaryngeal strap muscles to actively pull the cricoid cartilage forward, mechanically pulling the sphincter open [60]

## Cricopharyngeaus muscle dysfunction: pathophysiology

The pathophysiology of cricopharyngeus muscle dysfunction (CPMD) is still emerging. Current hypotheses assume CPMD is caused by an abnormal pathophysiologic swallowing mechanism failure resulting in limitation of relaxation of the cricopharyngeus with reduced bolus flow through the pharyngoesophageal segment [14]. Generally stated, CPMD refers to failed, diminished, or uncoordinated relaxation of the upper esophageal sphincter that *should* occur with normal swallowing. Cook described pathophysiology of the disorder within two categories: structural and functional [14].

Structural dysfunction relates specifically to what practitioners typically refer to as “CP bar”, a posterior protrusion on a lateral projection of a barium fluoroscopic study, defined to occur when an indentation more than 50% of the esophageal lumen is present [21]. However, as Cook discusses, presence of CP bar does not always result in dysphagia, but may contribute to symptoms in cases where other impairments exist, such as pharyngeal hypo-contractility. Further, histological indicators hypertrophy in the CP muscle do not always present clinically at a CP bar, and patients with CP bar have normal resting pressures as measured on manometry [14]. According to Cook, functional disorders include coordination of opening and true achalasia of the CP muscle, which can only be diagnosed with manometry, where the CP muscle fails to relax during the pharyngeal swallow [14]. When combined with other oropharyngeal impairments, such as reduced pharyngeal contraction or hyolaryngeal elevation and excursion, CP achalasia can result in a major obstruction to bolus flow, resulting in compromised swallow safety (i.e. increased risk of aspiration) and efficiency (i.e. presence of bolus material remaining in pharynx after the swallow).

Aside from radiation or surgical damage to the CP muscle resulting in structural dysfunction, it remains very unclear if CPMD is a result of muscular change (i.e. atrophy, hypertrophy) affecting muscular relaxation and compliance or resulting from issues related to coordination of neurologic signaling to the cricopharyngeal region [14]. Suspicions are high that pathophysiology of this disorder is likely a combination of biological makeup of the CP, oropharyngeal environment (i.e. how functional are other oral and pharyngeal mechanisms), and neurological innervation and signaling.

## Diagnosing cricopharyngeus muscular dysfunction

CPMD is a clinical diagnosis: patients presenting with obstructive symptoms and confirmed by either radiologic findings or manometry [21]. Typical course for diagnosis can present in 2 ways: formal workup for CPMD based on symptomology or incidental finding for workup of other oropharyngeal or pharyngoesophageal disorders. Patients presenting to primary care or otolaryngology with hallmark CPMD symptoms for CPMD dysfunction (e.g. globus sensation, feeling tightness during swallowing) trigger referrals and instrumentation to yield a diagnosis of CPMD. In some cases, CPMD may be an incidental finding discovered during a modified barium swallow study with a speech-language pathologist for suspected oropharyngeal dysphagia or pharyngoesophageal dysphagia assessments by a gastroenterologist (e.g. esophageal manometry, esophagram or esophagogastroduodenoscopy [EGD]). Given the interdisciplinary nature of these assessments, patients are often referred between laryngologists, speech-language pathologists, and gastroenterologists during differential diagnosis.

**Fig. 1:**
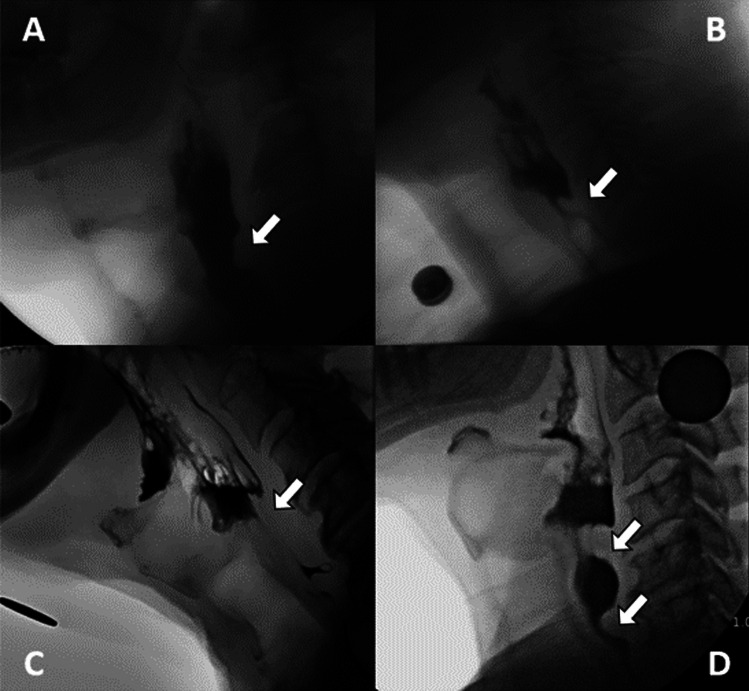
Examples of range of cricopharyngeal dysfunction at the level of the pharyngoesophageal segment. **A** Mild: distention is reduced, but flow is uninhibited; **B** Moderate: distention and flow are both impacted; **C** Severe: pharyngoesophagel segment + cricopharyngeal bar with possible early Zenker Diverticulum formation; **D** Double cricopharyngeal region impression with severe obstruction of bolus flow. Arrows indicate points of stricture

**Fig. 2:**
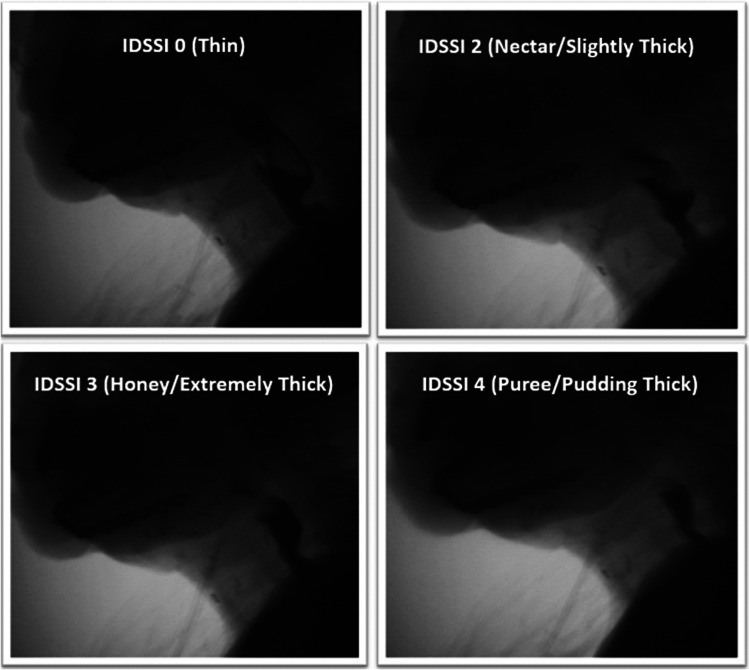
Examples of differences in cricopharyngeal impression by consistency presented under fluoroscopy during modified Barium Swallow Study (MBBS); *IDDSI* International Dysphagia Diet Standardization Initiative (Varibar terminology/IDDSI terminology)

Generally, when a patient presents with symptoms, videofluoroscopy is used to confirm CPMD diagnosis, with or without manometry [5, 22, 23]. The use of high-resolution pharyngeal manometry (HRPM) in the workup of pharyngeal dysphagia is an emerging tool that ultimately may prove superior in the ultimate diagnosis of CPMD. As the preliminary work of establishing normative values for HRPM, Walters et al. performed a meta analysis finding that both age and position alter both pharyngeal contractile measures and UES pressures [24]. However, the currently accepted instrumental evaluation for CPMD is fluoroscopy and fluoroscopy is highly varied depending on the degree of impairment. Figure 1 illustrates 4 different examples of both non-obstructive and obstructive impressions along the cervical esophagus, including mild CP impressions that do not obstruct bolus flow (Panel A), and ‘classic’ cricopharyngeal bars (Panel B, referred to clinically as “CP bar”) that obstruct bolus flow. There are also cases where there is narrowing of the pharyngoesophageal segment with or without a classic CP bar (e.g. Figure 1, Panel C: PES narrowing + bar) or cases where there is dynamic movement in areas of constriction throughout the upper esophagus causes multiple impressions during the swallow (Panel D). Since there is no universal method for classifying the degree of CP impression within the PES, Modified Barium Swallowing Impairment Profile (MBSImP) scoring can be used to characterize to what degree CPMD disrupts bolus flow, taking into account both distention and duration of opening of the pharyngoesophageal segment, which includes relaxation of the CP [25, 26]. According to this scale, any degree of reduced distention/duration/bolus obstruction is considered “abnormal” function. Further, certain consistencies highlight differences in CP dysfunction (examples in Fig. 2), which is why evidence-based protocols for fluoroscopy require additional textures [25, 26]. While this scale marks disruption of bolus flow through this region, there are no other criteria for determining severity of CPMD using videofluoroscopy or manometry.

While there are multiple ways and degrees of disorder, some patients may not be symptomatic to CPMD. If found incidentally, even if the patient is asymptomatic, it is important that CPMD is documented and diagnosed. Although the patient may not be symptomatic currently, the dysfunction may progress and result in worsening dysphagia or symptoms over time. While there is a poor understanding of the stability of CPMD vs progression over time, CPMD is implicated in other pharyngoesophageal issues (see below discussion about diverticula). Three are some cases where a patient presents with symptomology that align with possible CPMD, but no evidence of dysfunction on imaging or manometry. In these instances, continued follow-up and partnering between laryngologists, speech-language pathologists, and gastroenterologists is key for differential diagnosis and management of symptoms.

Another complicating factor is that the disorder may not only originate from CP dysfunction alone but can also stem from pharyngeal weakness; specifically with increasing dilation of the pharynx and increased pressure of the UES [27]. While some markers of pharyngeal weakness can be visualized on fluoroscopy (e.g. reduced pharyngeal stripping wave in lateral view and reduced A/P contraction, MBSImP components 12 and 13), the addition of high-resolution pharyngeal manometry (HRPM) can help illuminate specificity in the diagnoses by detailing pressure contributions of the pharynx through the upper esophagus [14]. However, again, no clear diagnostic criteria exist for determining a cut point for dysfunction using HRPM given high level of variability in published data and protocols [28]. One recent study suggested that patients with abnormal relaxation pressure of > 8 mmHg, measured using pharyngeal high-resolution manometry with impedance (HRPM-I), was a reliable marker of identifying patients with dysfunction of the UES as seen on videofluoroscopy [29]. However, there are no published guidelines or consensus on diagnostic criteria related to use of HRPM or HRPM-I. But in general, it is possible to measure UES tonic contraction and relaxation during a swallow (duration and timing), [30] which could theoretically aid in differential diagnosis of CP dysfunction in patients that may not consistently show obstruction on fluoroscopy.

## Special populations

### CPMD with diverticula

CPMD may occur with or without diverticula and causes a spectrum of disease [3, 31]. The most common CPMD with diverticula is a Zenker diverticulum (ZD). ZD is a pulsion type also called a pseudo-diverticulum, which is caused by herniation of mucosa and submucosa through a congenital dehiscence, called Killian’s triangle, between the oblique fibers of the inferior constrictor and the cricopharyngeus muscle [32]. It has been postulated that changes in muscular morphology, stemming from denervation or altered neurological input, contribute to development of CPMD with diverticula (i.e. Zenker diverticula) [33]. ZD involves CP obstruction and a congenital dehiscence described above causing a hypopharyngeal pouch. Interestingly, longitudinal studies of CPMD found stability of CP bar patients over time, but clinical worsening in ZD which is in direct contrast to the progressive theory of CPMD [3, 27].

**Fig. 3:**
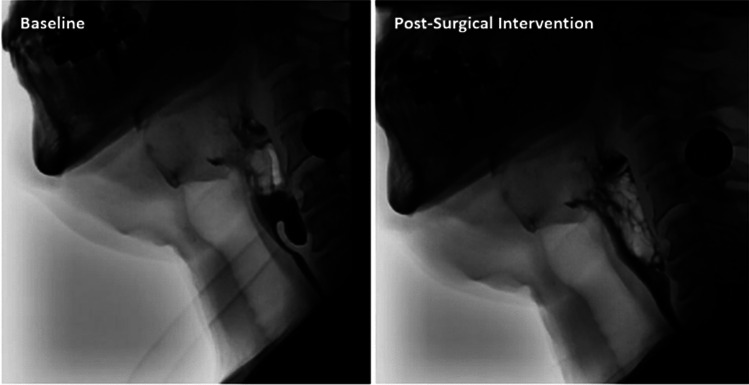
Zenker Diverticulum (ZD) before and after surgical management with an endoscopic cricopharyngeal myotomy with CO2 laser

ZD occur with an annual incidence of two per 100,000 patients; it should be clearly differentiated from the other hypopharyngeal diverticula (e.g., Killian Jamieson, Lamier, or iatrogenic diverticula) which may or may not be caused by CPMD because this effects the surgical treatment of choice (e.g., Killian Jamieson usually open approach given the relationship to the recurrent laryngeal nerve) [31, 32]. Treatment for ZD is surgical (see example, Fig. 3). Historically an open transcervical approach (still used today) by a variety of surgical techniques including inversion, diverticulopexy, and diverticulectomy. The transoral endoscopic methods were popularized in the 1980s and can be adapted using a variety of instruments including laser, stapler, flexible, or submucosal resection [34–37]. Most patients improve regardless of surgical approach, however most of the surgical literature is retrospective and single institution with broad variability and poorly defined outcome measures [38–40]. The POuCH Collaborative (11 institutions, prospective, multicenter, international database) reviewed both open and endoscopic techniques demonstrating that 66% of patients with ZD undergoing surgery will symptomatically improve in 66%, with 88% improving at least 50% in patient-reported dysphagia scoring with a modest advantage in transcervical open compared to transoral endoscopic technique. In another publication by the POuCH, open approaches do have increased complication rates and length of stay with no change in delays in oral intake [41, 42].

### Retrograde cricopharyngeus muscle dysfunction (RCPMD)

The upper esophageal sphincter normally reflexively relaxes second to transient lower esophageal relaxation and gaseous distension, termed the UES belch reflex [19]. The first case report of dysfunction of the belch reflex was described by Kahrilas et al. in 1987 with a 25-year-old woman with lifelong abelchia causing severe chest pain and abdominal discomfort, despite fluoroscopic and manometric normal swallowing physiology. However, both studies also demonstrated inability for UES relaxation with prolonged gas bubble distal to the UES two hours post-prandial and despite additional 30 ml air injection to the mid-esophagus [19, 43].

Retrograde cricopharyngeus muscle dysfunction (RCPMD) was first named by Bastian and Smithson in 1991 after successful intervention using high dose botulinum toxin A (botox) injection to the cricopharyngeus muscle [44]. The disorder typically effects men and women mean age 30 years (range 18–68) and clinical effectiveness persisted in majority after three months follow-up despite the typical wearing off effect of botox [45, 46]. Oude Nijhuis et.al prospectively enrolled 8 patients with symptoms of inability to belch performing manometry with belch provocation (drinking 500 mL carbonated water) and 24-h pH-impedance monitoring before and three months after UES botulinum toxin injection. These authors found normal UES relaxation with deglutition and continuous high-impedance indicating esophageal air entrapment which improved after treatment [47].

The clinical durability of botox treatment in RCPMD continues to be a pathophysiologic conundrum. Nonetheless, the increasing attention of this syndrome has been patient driven and self-identified often reading testimonials via social media, especially on internet platforms such as “Reddit” [48]. Increased patient awareness and self-advocacy with physicians’ willingness to learn has led to increased quality of life and dissemination of information [19, 48].

## Treatment

There is no agreed upon standard approach to either CPMD or CPMD with diverticula. While there are several approaches that are available, described in detail below, the order in which these treatment approaches should be applied, or which are most effective is still emerging. Whether or not patients are treated based on clinical presentation of symptoms, or presence and degree of CPMD impairment noted on instrumental evaluations is also practitioner-specific. Currently, treatment approaches are practice specific, and vary depending on the availability and training of physician partners and speech-language pathologists.

### Surgical management with otolaryngology

Several surgical options exist for treating CMPD, including dilation, botox injection, and cricopharyngeus myotomy [5, 22, 49, 50]. Success rates of each of these approaches have been examined, however reports are highly varied in the literature [49, 51]. One systematic review reports a weighted average of 78% success with myotomy vs 73% with dilation, compared to 69% success with botox injection [49]. Another review indicated that dilation provides temporary benefit, with a majority of patients undergoing repeat dilation or ultimately a CP myotomy [51]. However, the primary outcome measure used in this study was a subjective scale (Functional Outcome Swallowing Scale, FOSS) and not manometric or radiologic, which is a weakness of this study.

### Therapeutic management with speech-language pathology

Referral to a speech-language pathologist specializing in swallowing disorders for therapeutic management of CP dysfunction is common, although therapeutic approaches vary [52]. Exercises targeting hyolaryngeal suspension and excursion have been proposed and are commonly used, including the Shaker head lift exercise and the Mendelsohn maneuver [53, 54]. Although evidence varies on treatment efficacy [55]. More recently, it has been suggested that biofeedback therapy with HRPM could be beneficial for this particular physiological disorder, with the hope that patients can behaviorally modify the swallow to create better pressure transfer of the bolus through the UES [20, 56]. However, access to HRPM for clinical use by SLPs is limited [57].

## Remaining evidence gaps to be addressed

### Clear diagnostic criteria do not exist

The Prospective OUtcomes of Cricopharyngeus Hypertonicity (POUCH) Collaborative is a working group of international dysphagia-interested clinicians and researchers prospectively collecting a REDCap database since 2017 [31, 57, 58]. The multi-institutional, multi-disciplinary approach to CPMD through this group and others should expand on clear concise diagnostics will further our understanding of short-term interventions and long-term disability in this cohort of patients.

### Impact of CPMD on swallow safety, efficiency, and quality of life

Authors on this topic cited here agree that CP dysfunction is likely to increase aspiration and negatively impact swallow efficiency. However, there are no data available, to our knowledge, that describe how relative risk of swallow safety and efficiency is impacted with CPMD. As stated in the introduction, there is a poor understanding of prevalence rates for CPMD, [59] with high suspicion for under diagnosis. A clear understanding regarding impact of CPMD on swallow safety and physiology, specifically related to risk of aspiration and potentially life-threatening residue, is essential. Further, impact of CPMD on patient quality of life is even less well understood. Recent data from a prospective cohort study using the Eating Assessment Tool (EAT-10) patients with CPMD and Zenker diverticulae (ZD) presented with a mean (SD) score of 20.2(9.3) and 17.1 (10.1), respectively [19, 20], where a cut off of EAT-10 > 3 is indicative of abnormal impact on swallowing. These early data show that these disorders are highly impactful to patient quality of life. Future prospective and retrospective epidemiological studies of swallowing should focus on defining these risks and better understanding impact on patient experience, which will guide future investigative efforts and emphasize the importance of this area of research.

### Long-term effects of surgical intervention on CP muscle biology and physiology

While evidence exists regarding effects of cricopharyngeal myotomy on swallowing-related outcomes [51], there are few data to indicate how long dilation (with or without botox) will benefit a patient with symptomatic improvement, or any suggestion of the schedule for repeat dilations or injections [22]. Dilation with or without botox can be used as a precursor prior to myotomy to test whether the patient experiences symptomatic improvement, or if worsening reflux indicates a contraindication. There are no studies currently addressing the optimal order of surgical approaches: for example, should botox and/or dilation be attempted prior to myotomy in all cases, or are certain patient populations less likely to benefit from dilation, thus more appropriate for myotomy. Additionally, the biological impact of CP dilation on the anatomy, and ultimately physiology, of the cricopharyngeus muscle remains unclear. There are currently no available data on muscular effects of dilation of the CP muscle. Understanding what effects dilation has on muscle fiber structure, based on parameters and dilation approach (e.g. catheter size and type), is important to delineate to understand related physiological effects and how to maximize patient benefit from this procedure. These questions can only be addressed in well-designed prospective clinical trials examining these factors both short-term and longitudinally.

## Summary

Cricopharyngeus muscular dysfunction is an insidious disorder. Clinical symptomology and presentation are highly variable, diagnostic criteria are poorly defined, and treatment is equally as variable and poorly defined. Regardless of etiology, poor opening of the CP segment will more than likely negatively impact both patient-reported outcomes, diet tolerance, and safety. Impact of CP dysfunction can be devastating for patients and families, specifically relating to food avoidance and fear of choking, which are commonly reported in clinical experience. This is an area that is in dire need of well-designed, prospective and retrospective clinical trials to better define CPMD and understand the pathophysiology of available treatment approaches to improve quality of life for patients with CPMD.

## Data Availability

Not applicable.
